# Antigenic Subversion: A Novel Mechanism of Host Immune Evasion by Ebola Virus

**DOI:** 10.1371/journal.ppat.1003065

**Published:** 2012-12-13

**Authors:** Gopi S. Mohan, Wenfang Li, Ling Ye, Richard W. Compans, Chinglai Yang

**Affiliations:** 1 Department of Microbiology and Immunology, Emory University, Atlanta, Georgia, United States of America; 2 Department of Parasitology, Zhongshan School of Medicine, Sun Yat-sen University, Guangzhou, People's Republic of China; Mount Sinai School of Medicine, United States of America

## Abstract

In addition to its surface glycoprotein (GP_1,2_), Ebola virus (EBOV) directs the production of large quantities of a truncated glycoprotein isoform (sGP) that is secreted into the extracellular space. The generation of secreted antigens has been studied in several viruses and suggested as a mechanism of host immune evasion through absorption of antibodies and interference with antibody-mediated clearance. However such a role has not been conclusively determined for the Ebola virus sGP. In this study, we immunized mice with DNA constructs expressing GP_1,2_ and/or sGP, and demonstrate that sGP can efficiently compete for anti-GP_12_ antibodies, but only from mice that have been immunized by sGP. We term this phenomenon “antigenic subversion”, and propose a model whereby sGP redirects the host antibody response to focus on epitopes which it shares with membrane-bound GP_1,2_, thereby allowing it to absorb anti-GP_1,2_ antibodies. Unexpectedly, we found that sGP can also subvert a previously immunized host's anti-GP_1,2_ response resulting in strong cross-reactivity with sGP. This finding is particularly relevant to EBOV vaccinology since it underscores the importance of eliciting robust immunity that is sufficient to rapidly clear an infection before antigenic subversion can occur. Antigenic subversion represents a novel virus escape strategy that likely helps EBOV evade host immunity, and may represent an important obstacle to EBOV vaccine design.

## Author Summary

The function of the Ebola virus (EBOV) secreted glycoprotein (sGP) has been long debated, and the fact that sGP production is conserved among all known EBOV species strongly indicates an important role in the viral life cycle. Furthermore, the recent finding that EBOV mutates to a predominantly non-sGP-forming phenotype in cell culture, while the mutant virus reverts to an sGP-forming phenotype *in vivo*, suggests that sGP is critical for EBOV to survive in its infected host. Here we demonstrate that sGP can function to absorb anti-GP antibodies. More importantly, instead of simply passively absorbing host antibodies, sGP actively subverts the host immune response to induce cross-reactivity with epitopes it shares with membrane-bound GP_1,2_. Immune subversion by sGP represents a distinct mechanism from the use of secreted antigens as antibody decoys, an immune evasion tactic previously proposed for other viruses, and should be an important consideration for future EBOV vaccine design efforts since vaccines may need to be specifically tailored to avoid subversion.

## Introduction

Ebola virus (EBOV) is an enveloped single-stranded negative-sense RNA virus in the order *Mononegavirales*, which along with the Marburg virus (MARV) forms the Filovirus family. EBOV is the etiologic agent of Ebola Hemorrhagic Fever (EHF), a highly lethal hemorrhagic fever with up to 90% mortality [1]. Since its discovery in 1976, EBOV has caused sporadic outbreaks in Sub-Saharan Africa with death tolls in the hundreds. Interestingly, while filoviruses have been only recently discovered, they are one of the few non-retrovirus RNA paleoviruses identified in mammalian genomes, suggesting an ancient relationship with mammals [2], [3]. Growing evidence suggests that bats are the natural reservoir of EBOV in the wild today [4]–[6].

Current treatment for Ebola hemorrhagic fever is purely supportive, and the lack of effective interventions underscores the importance of developing a broadly-protective vaccine that confers long-lasting immunity. The ability to develop such a vaccine is critically dependent on our understanding of the mechanisms by which EBOV suppresses, distracts, or otherwise evades the host immune response [7]. One widely hypothesized immune evasion mechanism employed by Ebola virus is secretion of a truncated viral glycoprotein by EBOV infected cells. The EBOV surface glycoprotein (GP_1,2_) mediates host cell attachment and fusion, and is the primary structural component exposed on the virus surface. For this reason, GP_1,2_ is the focus of most EBOV vaccine research, and it is generally accepted that a robust anti-GP_1,2_ antibody response is crucial for protection against lethal EBOV challenge [8]. EBOV GP_1,2_ forms trimeric spikes on virion surfaces similarly to influenza HA and HIV Env [9]. Also like HA and Env, GP is first synthesized as an uncleaved precursor (GP_0_) which is then cleaved in the Golgi complex by the protease furin [10] into two functional subunits: The N-terminal GP_1_ subunit contains the putative receptor-binding domain (RBD), and the C-terminal GP_2_ subunit contains the fusion apparatus and transmembrane domain. GP_1,2_ is encoded in two disjointed reading frames in the virus genome. The two reading frames are joined together by slippage of the viral polymerase at an editing site (a tract of 7-A's) to insert an 8^th^ A, generating an mRNA transcript that allows read-through translation of GP_1,2_ [11], [12]. However, only about 20% of transcripts are edited, while the remaining 80% of unedited transcripts have a premature stop codon, resulting in synthesis of a truncated glycoprotein product (sGP) which is secreted in large quantities into the extracellular space.

Though its production is conserved in all EBOV species, there has been considerable debate regarding the function of sGP. Unlike GP_1,2_, sGP forms homodimers and appears to have some intrinsic anti-inflammatory activity [13]–[17]. The recent finding that EBOV quickly mutates to synthesize primarily GP_1,2_ in cell culture, while this mutant virus reverts to a primarily sGP-producing phenotype *in vivo*, suggests an important role for sGP in virus survival within the host [18]. Because sGP shares over 90% of its sequence with the N-terminal region of GP_1,2_, it was initially hypothesized that sGP functions as a decoy for anti-GP_1,2_ antibodies. Early efforts to identify such activity yielded mixed results, and the observation that antibodies often do not cross-react between sGP and GP_1,2_ had cast doubt on this hypothesis [19]–[23]. Furthermore, recent studies demonstrated that immunization against GP_1,2_ elicits antibodies largely against epitopes not shared with sGP [24]–[27]. However, most of these studies investigated monoclonal antibodies from animals immunized with vaccines containing or expressing primarily GP_1,2_, which does not represent the state of natural infection. Of note, one early study examined monoclonal antibodies from mice immunized with a Venezuelan equine encephalitis replicon that produces both GP_1,2_ and sGP, and found that many of these antibodies cross-reacted between GP_1,2_ and sGP [28]. Further, monoclonal antibodies isolated from human EHF survivors have been shown to preferentially react with sGP [19]. These studies suggest that sGP may play an important role in altering the host antibody response.

In this study, we demonstrate that sGP induces a host antibody response that focuses on epitopes it shares with GP_1,2_, thereby allowing it to bind and compete for anti-GP_1,2_ antibodies. We describe a mechanism that we term “antigenic subversion”, which is distinct from previously proposed “decoy” mechanisms in which secreted glycoprotein simply passively absorbs anti-glycoprotein antibodies. Importantly, we demonstrate that sGP can also subvert an existing anti-GP_1,2_ immune response that was only weakly cross-reactive with sGP. Antigenic subversion represents a novel host immune evasion mechanism that has important implications for EBOV vaccine design, and may shed light on how the virus survives in its natural reservoir.

## Results

### Immunogenicity of EBOV GP Editing Site Mutant DNA Vaccines

**Figure 1:**
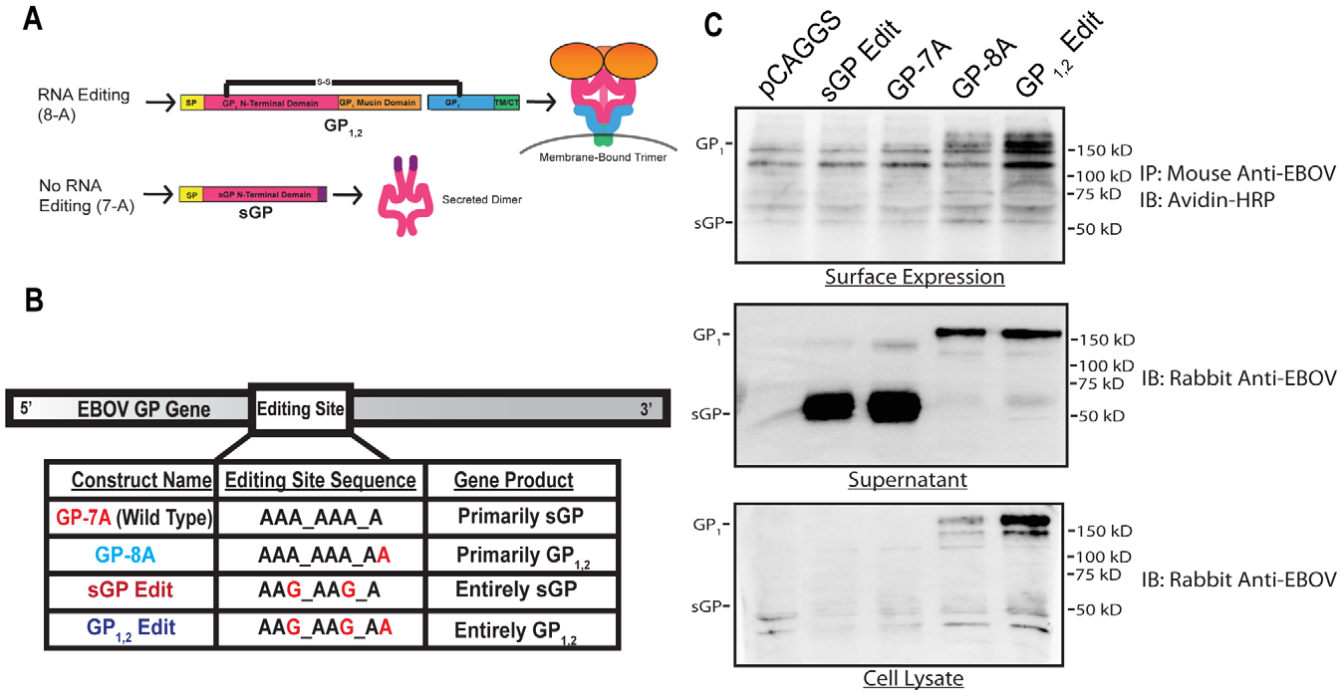
Diagram of EBOV RNA editing and construction of EBOV GP mutants. (A) Schematic diagram of GP_1,2_ and sGP. Membrane-bound GP_1,2_ is encoded in the EBOV genome in two disjointed reading frames. The GP editing site is a tract of 7 A's approximately 900 nucleotides downstream of the start codon. Slippage of EBOV RNA-dependent RNA polymerase at the editing site results in insertion of an 8^th^-A which brings the two GP reading frames in register resulting in read-through translation of full-length membrane-bound trimeric GP_1,2_. Unedited transcripts contain a premature stop codon and produce truncated dimerized sGP. (B) EBOV GP and editing site mutants. Mutated nucleotides are shown in red and the primary gene products expressed by these constructs are also listed. (C) Expression of EBOV GP by wild type and mutant DNA constructs. HeLa cells were transfected with the wild type GP or editing site mutant constructs and GP expression was assayed by Western blot at 48 h post-transfection.

We first generated EBOV GP constructs to individually express GP_1,2_ and sGP. In natural infection, EBOV directs the synthesis of sGP and GP_1,2_ through differentially edited mRNA transcripts (Fig. 1A). However, it has been observed that DNA-dependent RNA polymerases (DDRP) do not edit with the same efficiency as the EBOV RNA polymerase [12]. Furthermore, in addition to polymerase slippage, it is possible that the 7-A editing site can also serve as a premature poly-adenylation signal, as well as a ribosomal slippage signal [29]–[31]. We thus generated a panel of EBOV GP editing site mutants in order to control the levels of sGP and GP_1,2_ expression (Fig. 1B). GP-8A was made by inserting an 8^th^ A into the wild type (GP-7A) editing site, resulting in GP_1,2_ as the dominant gene product. Silent A→G mutations were introduced into the GP-8A editing site to ablate transcriptional slippage, resulting in GP_1,2_Edit, that expresses GP_1,2_ as the sole gene product. The same mutations were also introduced into GP-7A to generate sGPEdit, that expresses sGP as the sole gene product. These constructs were subcloned into a mammalian expression vector (pCAGGS) and protein expression was examined in both HeLa cells (Fig. 1C) and 293T cells (data not shown). Cells transfected with GP-8A and GP_1,2_Edit expressed GP_1,2_ intracellularly and on their surfaces, and secreted GP_1,2_ into the supernatant through previously characterized TACE-dependent cleavage [32]. Interestingly, GP_1,2_Edit produced higher amounts of GP_1,2_ than GP-8A. GP-7A and sGPEdit expressed high levels of sGP, which was secreted efficiently into the supernatant. GP_1,2_ expression by GP-7A was undetectable, likely because of minimal DDRP-mediated editing [12]. These expression experiments demonstrate that mutation of the editing site has a significant effect on GP expression.

**Figure 2:**
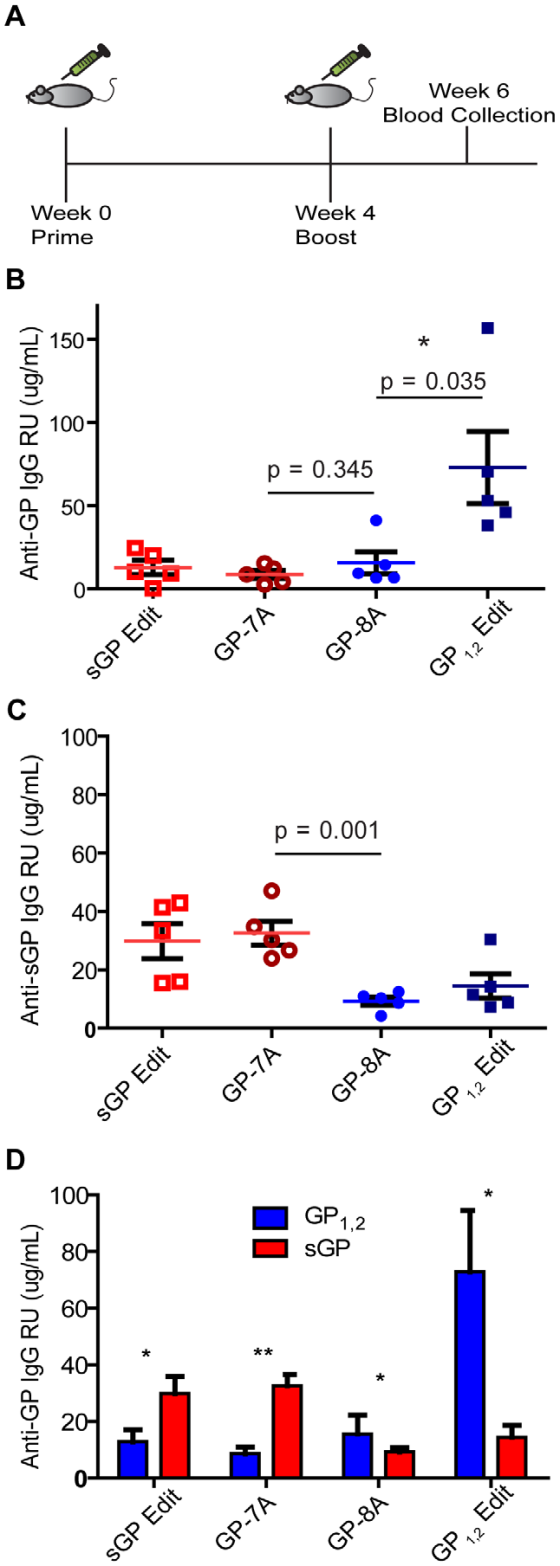
Immunogenicity of EBOV GP editing site mutants. (A) Immunization study design. Female BALB/C mice were immunized with the four editing site mutant constructs in the pCAGGS vector. Mice were vaccinated IM with 50 µg of DNA (25 µg/leg) according to the schedule shown. (B) Antibody response against GP_1,2_. (C) Antibody response against sGP. The levels of antibody response induced by EBOV GP DNA constructs in mice were measured by ELISA using His-GP_1,2_ or His-sGP as coating antigen. Antibody concentration was determined from a standard curve and expressed as µg/mL of anti-GP IgG. Asterisks indicate statistically significant difference between groups and P-values are given in red. (D) Comparison of antibody levels against GP_1,2_ and sGP induced by each EBOV GP DNA construct. Average titers of anti-GP_1,2_ (blue) and anti-sGP (red) antibodies within immunization groups are shown for comparison of the GP isoform reactivity profiles both within and between immunization groups. Asterisks indicate statistically significant differences between anti-GP_1,2_ and anti-sGP titers within groups, as measured by paired, two-tailed Student's t-test (* = p<0.05, ** = p<0.001).

We next investigated the immunogenicity of editing site mutant DNA vaccines. Female BALB/c mice were immunized with GP_1,2_ or sGP-producing constructs (Fig. 2A). Mice immunized with sGPEdit, GP-7A, and GP-8A constructs developed similar titers of anti-GP_1,2_ antibodies as measured by ELISA, while mice immunized with GP_1,2_Edit developed four-fold higher titers of anti-GP_1,2_ antibodies (Fig. 2B). Mice immunized with constructs expressing predominantly sGP (GP-7A and sGPEdit) developed much higher titers of anti-sGP antibodies than mice immunized with constructs expressing predominantly GP_1,2_ (GP-8A or GP_1,2_Edit) (Fig. 2C). As shown in Fig. 2D, GP_1,2_-immunized mice developed much higher titers of GP_1,2_-binding antibodies than sGP-binding antibodies. On the other hand, sGP-immunized mice developed much higher titers of sGP-binding antibodies than GP_1,2_-binding antibodies, despite the fact that sGP shares roughly 95% of its linear sequence with GP_1,2_. These results suggest that in sGP-immunized animals, either many sGP-binding antibodies are directed against conformational epitopes not shared with GP_1,2_, or they are directed against shared epitopes that are inaccessible in GP_1,2_.

### sGP Can Compete for Binding of Anti-GP_1,2_ Antibodies Induced by sGP but not by GP_1,2_

**Figure 3:**
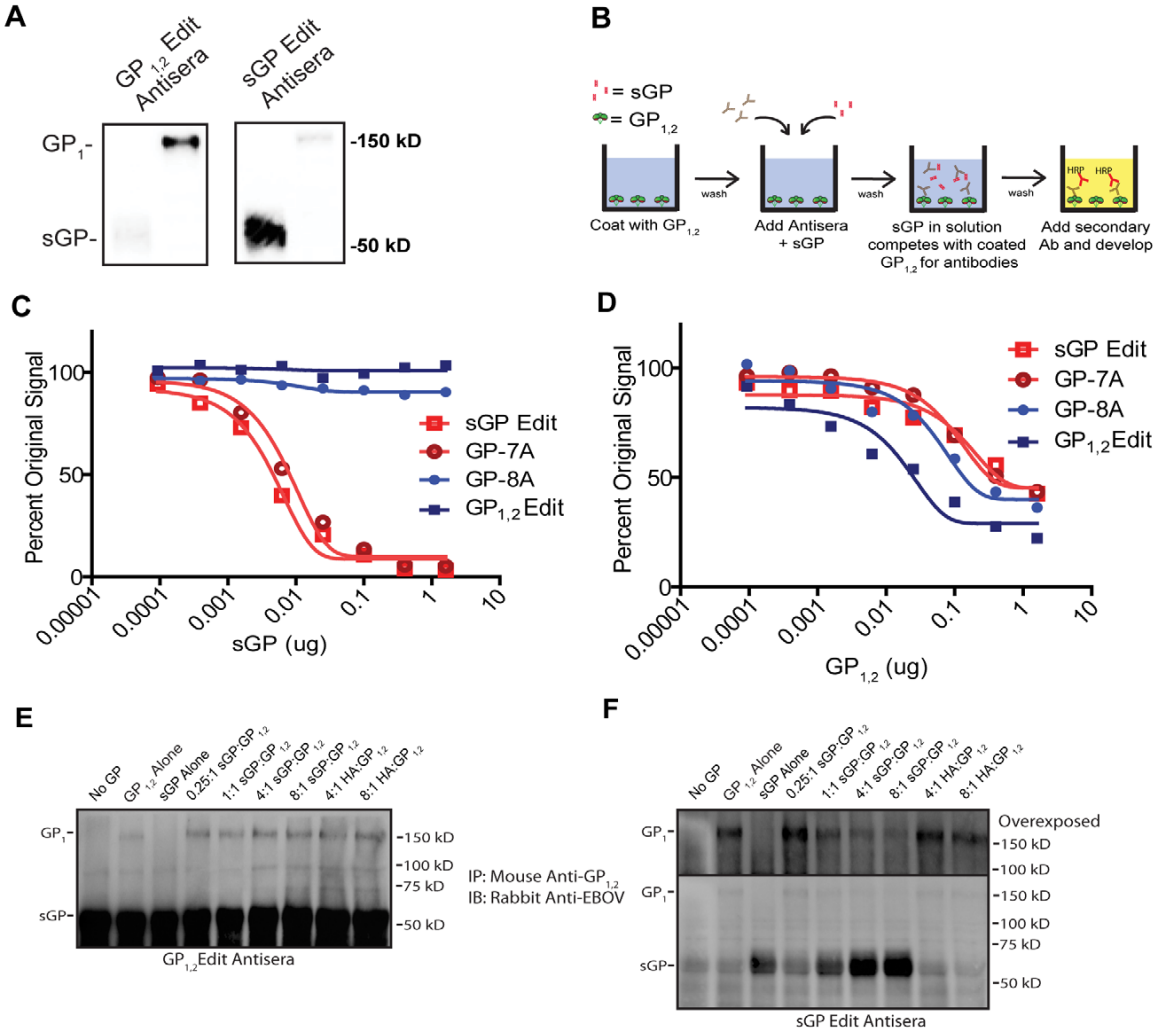
Antiserum from mice immunized against GP_1,2_ or sGP display different reactivity patterns. (A) Detection by Western blot of antibodies against GP_1,2_ and sGP from immunized mice. 50 ng of purified His-sGP and His-GP_1,2_ were run by SDS-PAGE under denaturing conditions and probed with 1∶1000 pooled GP_1,2_Edit or sGPEdit antisera followed by blotting with HRP-conjugated goat anti-mouse IgG. (B) Schematic of competition ELISA. Wells were coated with GP_1,2_ and incubated with pooled antisera as well as increasing concentrations of competing antigen (sGP or GP_1,2_) to compete for antibodies. After two hours, plates were washed and then incubated with HRP-conjugated secondary antibody followed by addition of substrate to develop color. (C, D) Competition ELISA. Antisera from mice immunized with sGPEdit, GP-7A, GP-8A, and GP_1,2_Edit were diluted to give similar anti-GP_1,2_ signal. Diluted antiserum was mixed with increasing quantities of purified His-sGP (C) or His-GP_1,2_ (D) and incubated in His-GP_1,2_ coated wells and developed as described above. Experiments were performed in duplicate and repeated at least three times, with representative results shown. (E, F) Competition Immunoprecipitation. Pooled antisera from GP_1,2_Edit-immunized mice (E) or sGP-immunized mice (F) were incubated with no GP, purified sGP or GP_1,2_ alone, or with fixed GP_1,2_ and increasing concentrations of sGP to compete for anti-GP_1,2_ antibodies. GP_1,2_ was incubated with recombinant HA as a negative control. The upper panel for the sGPEdit antisera shows the GP_1,2_ portion of the blot at a longer exposure time to show the attenuation of signal with increasing sGP concentration. Results are representative of three independent experiments.

Given that animals immunized by GP_1,2_ or sGP develop antibodies that preferentially bind to different GP isoforms, we performed Western blot analysis to determine if there is a difference in the linear epitopes targeted by antibodies in GP_1,2_ versus sGP-immunized mice. As shown in Fig. 3A, antisera from GP_1,2_-immunized mice reacted strongly with GP_1,2_ but only weakly with sGP. On the other hand, antisera from sGP-immunized mice reacted strongly with sGP, but only weakly with GP_1,2_. This suggests that most linear epitopes targeted by anti-GP_1,2_ antibodies from GP_1,2_-immunized mice are unshared with sGP. To investigate whether the GP_1,2_-binding and sGP-binding antibodies in immunized mice were cross-reactive between the two GP isoforms or were separate populations of antibodies, we performed a competition ELISA to determine if sGP could compete with GP_1,2_ for GP_1,2_-binding antibodies (Fig. 3B). Similar to the Western blot data, sGP was unable to compete for binding of anti-GP_1,2_ antibodies from GP_1,2_ immunized mice (Fig. 3C). On the other hand, sGP was able to efficiently compete for anti-GP_1,2_ antibodies from sGP-immunized mice. As expected, GP_1,2_ was able to compete with itself in all groups (Fig. 3D). Furthermore, we observed an identical reactivity pattern with native membrane-anchored EBOV GP_1,2_ using a cell surface competition ELISA (Supplemental Fig. S1). We further examined the ability of the two GP isoforms to compete with each other for antibodies by performing competition immunoprecipitation. Purified GP_1,2_ in the presence of sGP at varying molar ratios was immunoprecipitated with antiserum from GP_1,2_-immunized or sGP-immunized mice, and analyzed by Western blot using a polyclonal rabbit antibody that reacts with both GP isoforms. Antiserum from GP_1,2_-immunized mice precipitated both GP_1,2_ and sGP, and increasing concentrations of sGP did not attenuate the amount of GP_1,2_ signal (Fig. 3E), suggesting the presence of two separate populations of antibodies that do not cross-react between GP_1,2_ and sGP. However, while antiserum from sGP-immunized mice also precipitated both GP_1,2_ and sGP, increasing concentrations of sGP significantly attenuated the amount of GP_1,2_ precipitated (Fig. 3F), indicating that GP_1,2_-reactive antibodies in these mice are cross-reactive with sGP. As a control, addition of recombinant HA had no effect on the amount of GP_1,2_ precipitated by either antiserum group. Taken together, these data suggest that anti-GP_1,2_ antibodies induced by GP_1,2_ are directed primarily against epitopes not shared between GP_1,2_ and sGP, whereas such antibodies induced by sGP are directed against epitopes shared between GP_1,2_ and sGP.

### sGP Differentially Interferes with Antibody-mediated Viral Neutralization by Antisera from sGP and GP_1,2_ Immunized Mice

**Figure 4:**
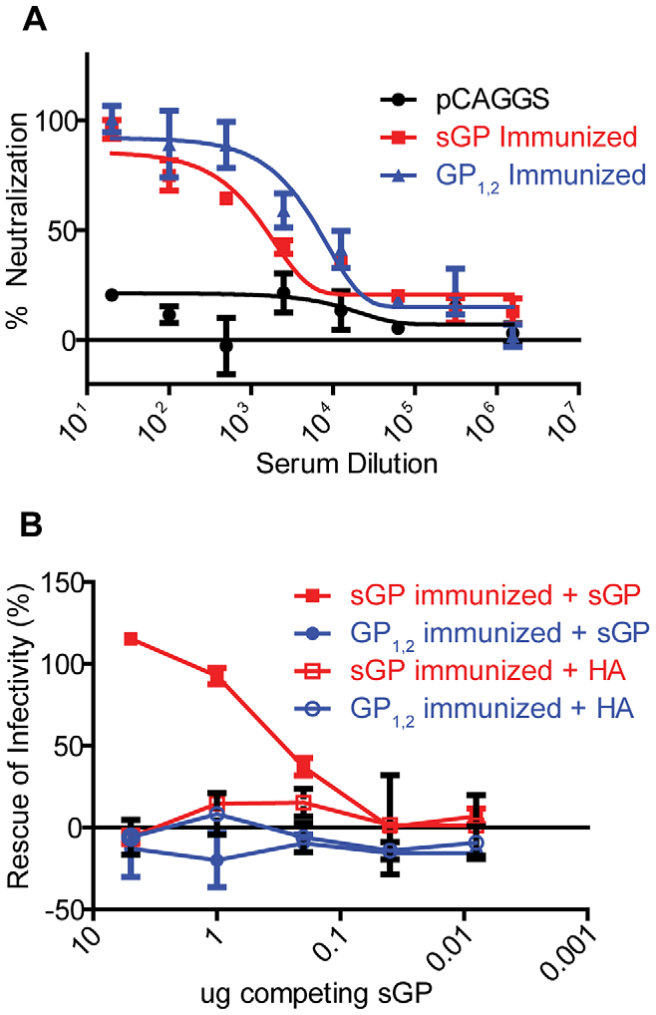
Interference with antibody-dependent neutralization by sGP. (A) Neutralization of EBOV GP pseudovirus. Neutralizing activity of antisera was determined by incubating 500 pfu of GP_1,2_-pseudotyped virus with dilutions of pooled GP_1,2_-immunized (Blue), sGP-immunized (Red), and empty pCAGGS vector-immunized (black) antisera. Neutralization was measured as decrease in luciferase expression compared to virus-only controls after 48 h. (B) Interference of EBOV GP pseudovirus neutralization by sGP. The ability of sGP to interfere with antibody-dependent neutralization was determined by allowing sGP to compete with GP_1,2_ pseudotyped viruses for anti-GP_1,2_ antibodies. Pooled GP_1,2_-immunized (blue) and sGP-immunized (red) antisera were fixed at the dilution corresponding to 80% neutralization. Antisera was co-incubated with increasing dilutions of His-tagged sGP (solid markers) or His-tagged influenza PR8 HA (open markers), and rescue of infectivity was measured as described in methods.

We further investigated whether there was a difference in the ability of antisera from the immunization groups to neutralize EBOV GP_1,2_-mediated virus infection, and whether sGP could interfere with antibody-mediated neutralization. Pseudoviruses were generated using an Env-deficient HIV backbone pseudotyped with Zaire EBOV GP_1,2_. In order to achieve consistent neutralization, we pooled sera from the four highest responders among GP_1,2_-immunized animals and among sGP-immunized animals. Antisera from both groups were able to effectively neutralize pseudoviruses as measured by a luciferase reporter assay (Fig. 4A), although antisera from GP_1,2_-immunized mice exhibited more potent neutralizing activity than antisera from sGP-immunized mice, probably due to higher overall anti-GP_1,2_ titer. To determine if sGP interferes with neutralization, we used an antiserum dilution corresponding to 80% neutralizing activity in each group and preincubated antisera with different amounts of sGP. Consistent with the competition ELISA results, sGP was able to completely attenuate neutralizing activity of antisera from sGP-immunized mice, while it had no effect on neutralizing activity of antisera from GP_1,2_-immunized mice (Fig. 4B). Purified influenza HA was used as a control and had no effect on neutralizing activity of either antiserum group. Similar results were observed when we used an antiserum dilution corresponding to 50% neutralizing activity (Supplemental Fig. S2). These data confirm that sGP can compete with GP_1,2_ for anti-GP_1,2_ antibodies and interfere with antibody-mediated neutralization, but can only do so in animals that have been exposed to sGP.

### Anti-GP_1,2_ and Anti-sGP Antibodies Induced by Different GP Isoforms Exhibit Similar Average Affinity

**Figure 5:**
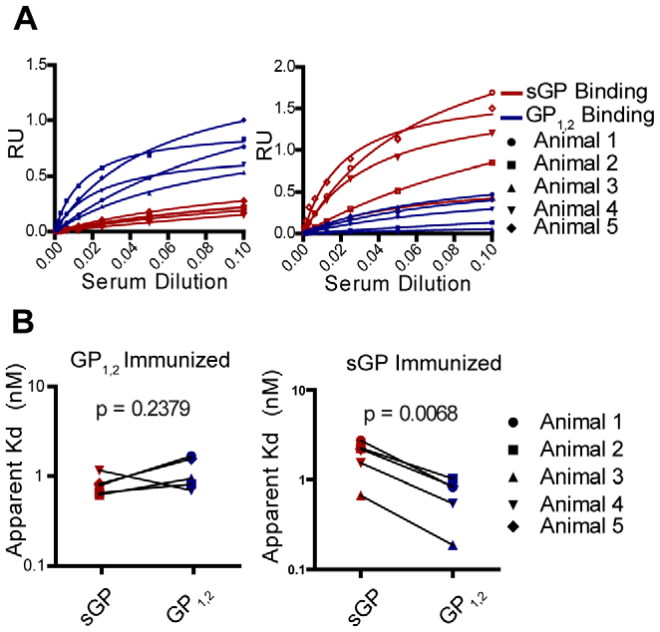
Comparison of binding affinity of GP_1,2_-immunized versus sGP-immunized antisera for sGP and GP_1,2_. (A) Determining apparent K_d_ value of antibodies from immunized mice for GP_1,2_ and sGP. Antiserum from five mice immunized against GP_1,2_ and five mice immunized against sGP were individually analyzed by quantitative ELISA using GP_1,2_ (blue) or sGP (red) as coating antigen. Scatchard analysis was used to calculate apparent dissociation constants (K_d_). (B) Comparison of antibody affinity for GP_1,2_ and sGP. Comparison of apparent K_d_'s of GP_1,2_-immunized and sGP-immunized polyclonal antisera for sGP (red) and GP_1,2_(blue) was determined by nonlinear regression analysis of Scatchard plots. K_d_'s for sGP and GP_1,2_ were calculated for five individual mice in each group and values for the same animal are connected by a black line.

The inability of sGP to compete with GP_1,2_ for antibodies from GP_1,2_-immunized mice was intriguing considering that GP_1,2_ shares almost half of its ectodomain sequence with sGP. We reasoned that some of these antibodies may be directed solely against GP_1,2_ epitopes not shared with sGP, while other antibodies may be directed against shared epitopes, but preferentially bind GP_1,2_ because of conformational differences between the two GP isoforms resulting from tertiary and quarternary structure and steric shielding. To address this possibility, we used quantitiative ELISA to determine the relative titers and estimate the average affinity of antibodies from GP_1,2_ and sGP-immunized animals for GP_1,2_ and sGP. We individually examined purified polyclonal IgG from the five highest responders in GP_1,2_-immunized and sGP-immunized groups, and calculated the apparent dissociation constant (K_d_) of anti-GP_1,2_ and anti-sGP antibodies. This apparent K_d_ was calculated by Scatchard analysis as described elsewhere [33], [34] and represents an estimate of the average affinity of anti-GP antibodies, with lower apparent K_d_ correponding to higher average affinity. Consistent with above ELISA data (Fig. 2D), mice immunized against GP_1,2_ had higher titers of anti-GP_1,2_ antibodies than anti-sGP antibodies (Fig. 5A). However, there was no measurable difference in the apparent K_d_'s of GP_1,2_-binding vs. sGP-binding antibodies (Fig. 5B), indicating that preferential binding of antibodies from these animals to GP_1,2_ is not due to affinity differences for different GP isoforms. In mice immunized against sGP we again observed very high titers of anti-sGP antibodies, and very low levels of anti-GP_1,2_ antibodies. However, those antibodies that did bind to GP_1,2_ appeared to have modestly lower K_d_ (higher average affinity) than did sGP-binding antibodies (Fig. 5B). Future studies with monoclonal antibodies directed against epitopes shared between sGP and GP_1,2_ will provide further information on whether specific antibodies bind to the two GP isoforms with different affinities. Nonetheless, the present data provide evidence that differences in affinity are not responsible for antibodies from GP_1,2_ and sGP-immunized mice reacting preferentially with different GP isoforms.

### Expression of GP_1,2_ in the Context of sGP Allows sGP to Compete for Anti-GP_1,2_ Antibodies

**Figure 6:**
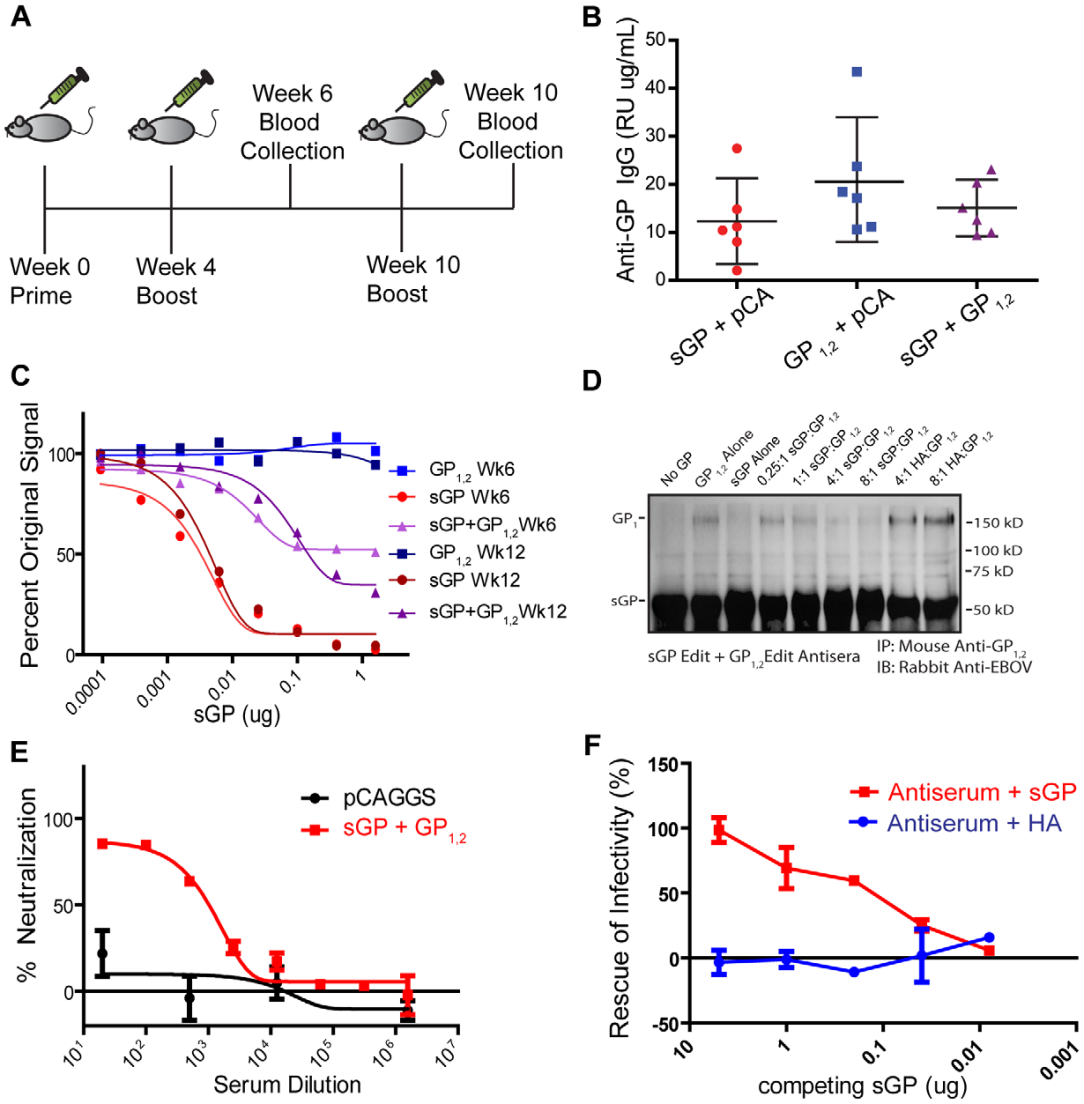
The effect of sGP on immune response when antigen exposure mimics natural infection. (A) Immunization study design. Female BALB/C mice were immunized IM with 50 µg of total DNA per immunization according to the schedule shown. Mice were immunized with a 3∶1 ratio of sGP Edit∶GP_1,2_ Edit in pCAGGS. Control groups were immunized with sGP Edit or GP_1,2_ Edit alone plus empty pCAGGS vector to keep total amount of immunizing DNA constant. (B) Comparison of antibody response against GP_1,2_. Mouse sera collected at week 6 were analyzed for anti-GP_1,2_ antibodies by ELISA using GP_1,2_ as coating antigen. (C) sGP competition ELISA. The ability of sGP to compete for anti-GP antibodies was determined by competition ELISA as in Figure 3B. Pooled antisera were analyzed from mice immunized with a GP_1,2_ Edit (blue), sGP Edit (red), or a 3∶1 ratio of sGP Edit∶GP_1,2_Edit (purple), and were diluted to give roughly equivalent anti-GP_1,2_ signal. Competition ELISA was performed from antisera collected at both week 6 (light color) and week 12 (dark color) according to the immunization schedule. (D) Competition immunoprecipitation. Pooled antisera from sGPEdit+GP_1,2_Edit-immunized mice were incubated with no GP, purified sGP or GP_1,2_ alone, or with fixed GP_1,2_ and increasing concentrations of sGP to compete for anti-GP_1,2_ antibodies. GP_1,2_ was incubated with recombinant HA as a negative control, and precipitated and analyzed as in Figure 3E,F. (E) Neutralization of EBOV GP pseudovirus. Neutralizing activity of antisera was determined by incubating 500 pfu of GP_1,2_-pseudotyped virus with dilutions of pooled sGP+GP_1,2_-immunized (red), or empty pCAGGS vector-immunized (black) antisera. Neutralization was measured as decrease in luciferase expression compared to virus-only controls. (F) Interference of EBOV GP pseudovirus neutralization by sGP. The ability of sGP to interfere with antibody-dependent neutralization was determined as in Figure 4B. Pooled sGP+GP_1,2_-immunized antisera were fixed at the dilution corresponding to 80% neutralization. Antisera were co-incubated with increasing dilutions of purified sGP (red) or purified influenza PR8 HA (blue), and rescue of infectivity was measured as described in methods.

The secretion of surface glycoproteins as a mechanism of absorbing antiviral antibodies has been hypothesized before for several viruses including vesicular stomatitis virus (soluble G) and respiratory syncytial virus (secreted G) [35], [36]. It has been demonstrated that RSV secreted G can absorb anti-G antibodies and interfere with both neutralization and antibody-dependent cell-mediated virus clearance. However, we observed that EBOV sGP can only compete for anti-GP_1,2_ antibodies in mice immunized against sGP. This led us to hypothesize that sGP may serve a role in altering the repertoire of epitopes against which the host immune response is directed, in order to divert the host immune response towards epitopes shared between sGP and GP_1,2_. To test this hypothesis, we vaccinated mice with a 3∶1 ratio of sGPEdit∶GP_1,2_Edit (Fig. 6A) to simulate antigen expression during EBOV infection. Control groups were immunized with either sGPEdit or GP_1,2_Edit plus empty pCAGGS vector to keep the total amount of DNA constant. As a proxy for *in vivo* antigen expression, HeLa cells were transfected with corresponding ratios of sGPEdit, GP_1,2_Edit, and pCAGGS. As measured by Western blot analysis, the levels of sGP and GP_1,2_ expression in both lysate and culture supernatant of cells co-transfected with sGPEdit and GP_1,2_Edit were similar to cells transfected with sGPEdit or GP_1,2_Edit alone (Fig. S3). All immunization groups generated similar titers of anti-GP_1,2_ antibodies (Fig. 6B). However, when we performed a competition ELISA using antisera from sGPEdit+ GP_1,2_Edit-immunized mice, sGP was able to compete with GP_1,2_ for over 50% of the anti-GP_1,2_ antibodies (Fig. 6C). Mice immunized with GP_1,2_Edit+vector or sGPEdit+vector displayed the same serum reactivity patterns we had observed previously in mice immunized against only one of the GP isoforms. Further, after boosting mice a second time, almost 70% of GP_1,2_-antibodies in week 12 antisera from sGPEdit+ GP_1,2_Edit-immunized mice were absorbed by sGP. Interestingly, in mice immunized with lower ratios of sGPEdit∶GP_1,2_Edit, significant sGP cross-reactivity was also observed, with almost 70% of anti-GP_1,2_ antibodies being susceptible to competition in mice immunized with a 1∶1 ratio of sGP∶GP_1,2_, and about 25% being susceptible to competition in mice immunized with a 1∶3 ratio of sGP∶GP_1,2_ (Figure S4). Similar results were also obtained with a competition immunoprecipitation assay. As shown in Fig. 6D, antiserum from sGPEdit+GP_1,2_Edit-immunized mice was able to precipitate both GP_1,2_ and sGP, but increasing concentrations of sGP attenuated the amount of GP_1,2_ precipitated. Furthermore, while sGPEdit+GP_1,2_Edit antiserum was able to effectively neutralize pseudovirus infectivity (Fig. 6E), the addition of exogenous sGP almost completely inhibited pseudovirus neutralization (Fig. 6F), indicating that sGP can effectively interfere with antibody mediated neutralization in these mice. Similar observations were also made at an antiserum concentration corresponding to 50% neutralization (Fig. S5). Taken together, these data confirm that sGP can direct the host antibody response to focus on epitopes shared between GP_1,2_ and sGP, thereby allowing sGP to compete for antibodies and interfere with antibody-mediated virus neutralization. Furthermore, the observation that sGP can compete for a greater proportion of GP_1,2_ antibodies from week 12 antisera compared to week 6 suggests that iterative exposure to sGP gradually drives the host to a dominantly sGP-reactive response.

### sGP Can Subvert the GP_1,2_-specific Antibody Response

**Figure 7:**
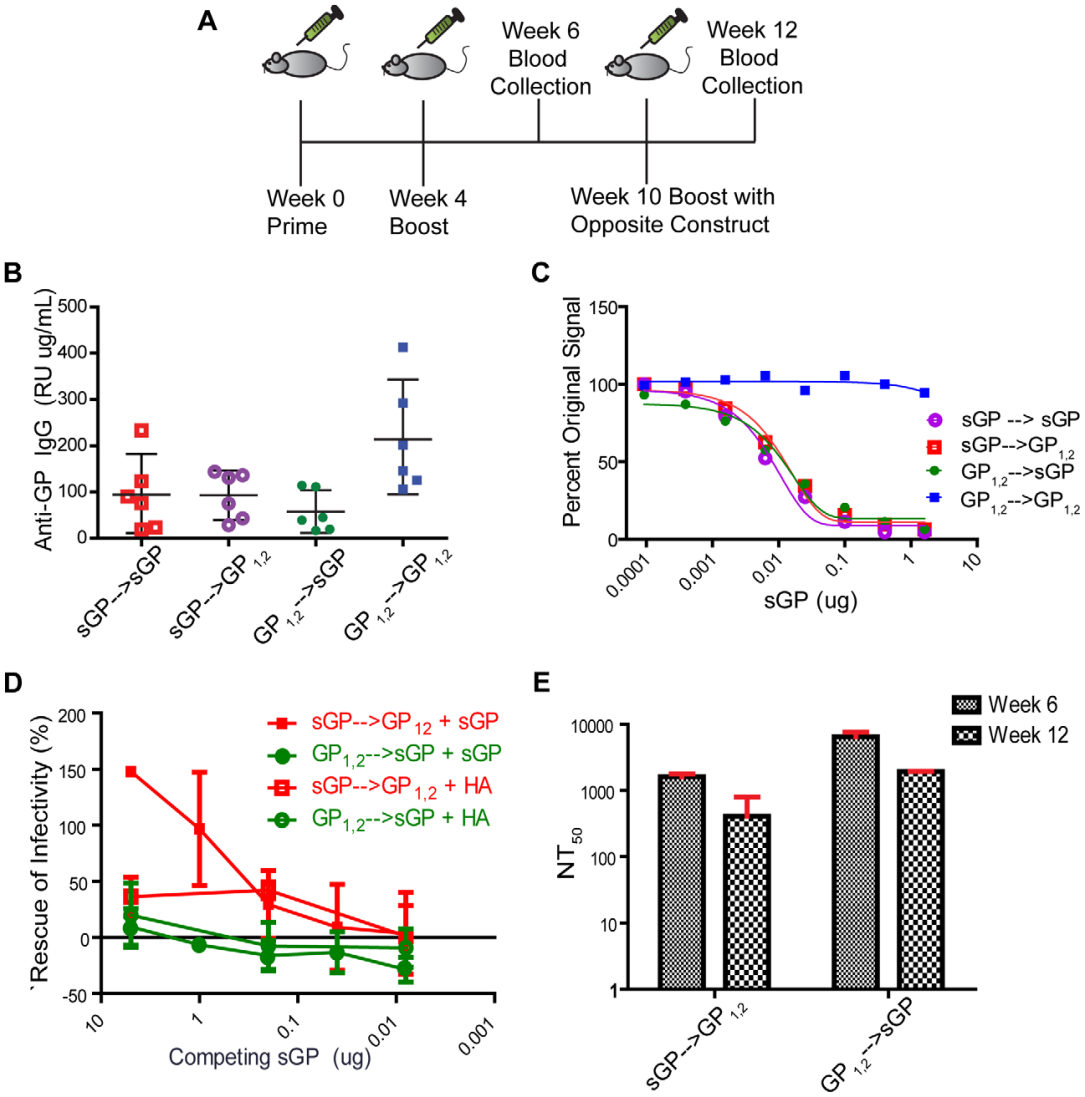
Ability of sGP to divert antibody responses against GP_1,2_. (A) Immunization study design. Female BALB/C mice were immunized IM with 50 µg of total DNA per immunization according to the schedule. Two groups of mice (n = 12) were primed and boosted as in previous experiments with either sGP Edit or GP_1,2_ Edit in pCAGGS vector. Each group was divided in two and subgroups were boosted at week 10 with either the same construct against which they had initially been immunized, or with the opposite editing site mutant construct. (B) Comparison of antibody response against GP_1,2_. Sera collected at week 12 were analyzed for antibodies against GP_1,2_ by ELISA using GP_1,2_ as coating antigen. (C) sGP competition ELISA. The ability of sGP to compete for anti-GP_1,2_ antibodies was determined by competition ELISA as described in Figure 3B. Pooled antisera were analyzed from mice immunized with sGP Edit and then boosted at week 10 with either GP_1,2_ Edit (red), or sGP Edit (purple), and from mice immunized with GP_1,2_ Edit and then boosted at week 10 with either GP_1,2_Edit (blue) or sGP Edit (green). All ELISA experiments were performed in duplicate at least three times and representative results shown. (D) Interference of EBOV GP pseudovirus neutralization by sGP. The ability of sGP to interfere with antibody-dependent neutralization was determined as in Figure 4B. Pooled sGP-primed, GP_1,2_-boosted (red) and GP_1,2_-primed, sGP-boosted (green) antisera were fixed at the dilution corresponding to 50% neutralization. Antisera were co-incubated with increasing dilutions of His-tagged sGP (solid markers) or His-tagged influenza PR8 HA (open markers), and rescue of infectivity was measured as described in methods. (E) Comparison of 50% neutralization titers. Antiserum titers corresponding to 50% pseudovirus neutralization activity (NT_50_) were calculated for week 6 (fine checkered) and week 12 (coarse checkered) mice. Error bars correspond to 95% confidence interval as determined by Student's t-test.

In order to test the hypothesis that expression of sGP can modulate the GP_1,2_-specific antibody response, we primed and boosted mice with either sGPEdit or GP_1,2_Edit, and then boosted again at week 10 with the opposite GP isoform (Fig. 7A). Control groups were boosted with the same GP isoform. As shown in Fig. 7B, anti-GP_1,2_ antibodies were induced in all groups at week 12. However, in mice immunized with GP_1,2_Edit and then boosted with sGPEdit, sGP was able to efficiently compete for anti-GP_1,2_ antibodies in competition ELISA (Fig. 7C). Furthermore, sGP was also able to efficiently compete for anti-GP_1,2_ antibodies from mice primed against sGPEdit and boosted with GP_1,2_Edit. We next investigated whether sGP is able interfere with virus neutralization by sera from cross primed and boosted mice. As shown in Fig. 7D, sGP was able to interfere with neutralization only from animals primed against sGP and boosted with GP_1,2_. On the other hand, antisera from animals primed against GP_1,2_ and boosted with sGP maintained their neutralizing activity in the presence of sGP. To further probe this observation, we compared the antisera titers corresponding to 50% neutralizing activity (NT_50_) in groups before (week 6) and after (week 12) boosting with the opposite GP isoform. As shown in Fig. 7E, neutralizing activity is not boosted by immunization with the opposite GP isoform. Thus, it appears not only that sGP can overwhelm the GP_1,2_-specific response, but also that it only boosts non-neutralizing antibodies induced by GP_1,2_. The observation that sGP can alter the reactivity profile of the anti-GP_1,2_ response has important implications for EBOV vaccinology, since during a infection, sGP could subvert the immune response of a previously vaccinated individual if the virus is not cleared rapidly.

## Discussion

The role of sGP in EBOV host immune evasion has not been clearly defined. In this study, we analyzed antibody responses in mice immunized against sGP, GP_1,2_, or both GP isoforms and present evidence that sGP serves to redirect the immune response towards epitopes that are either not present or inaccessible in GP_1,2,_ or epitopes that are shared between the two GP isoforms, thereby allowing sGP to effectively absorb anti-GP_1,2_ antibodies. We term this phenomenon “antigenic subversion”, because it is distinct from previously proposed mechanisms in which sGP passively absorbs anti-glycoprotein antibodies. In antigenic subversion, the ability of sGP to absorb anti-GP_1,2_ antibodies is critically dependent on exposure to sGP during induction of the anti-GP_1,2_ immune response. In mice immunized against GP_1,2_ in the presence of sGP, an immunization strategy designed to simulate antigen exposure during natural infection, we observed that most resulting anti-GP_1,2_ antibodies were cross reactive with and thus susceptible to competition by sGP, even though the titers of anti-GP_1,2_ antibodies in these mice were similar to the titers in mice immunized against GP_1,2_ alone. On the other hand, in mice immunized against GP_1,2_ alone, we observed only low cross-reactivity of anti-GP_1,2_ antibodies with sGP, a finding consistent with previous studies, indicating that antibodies in these mice are largely directed against epitopes not shared with sGP [23], [24].

**Figure 8:**
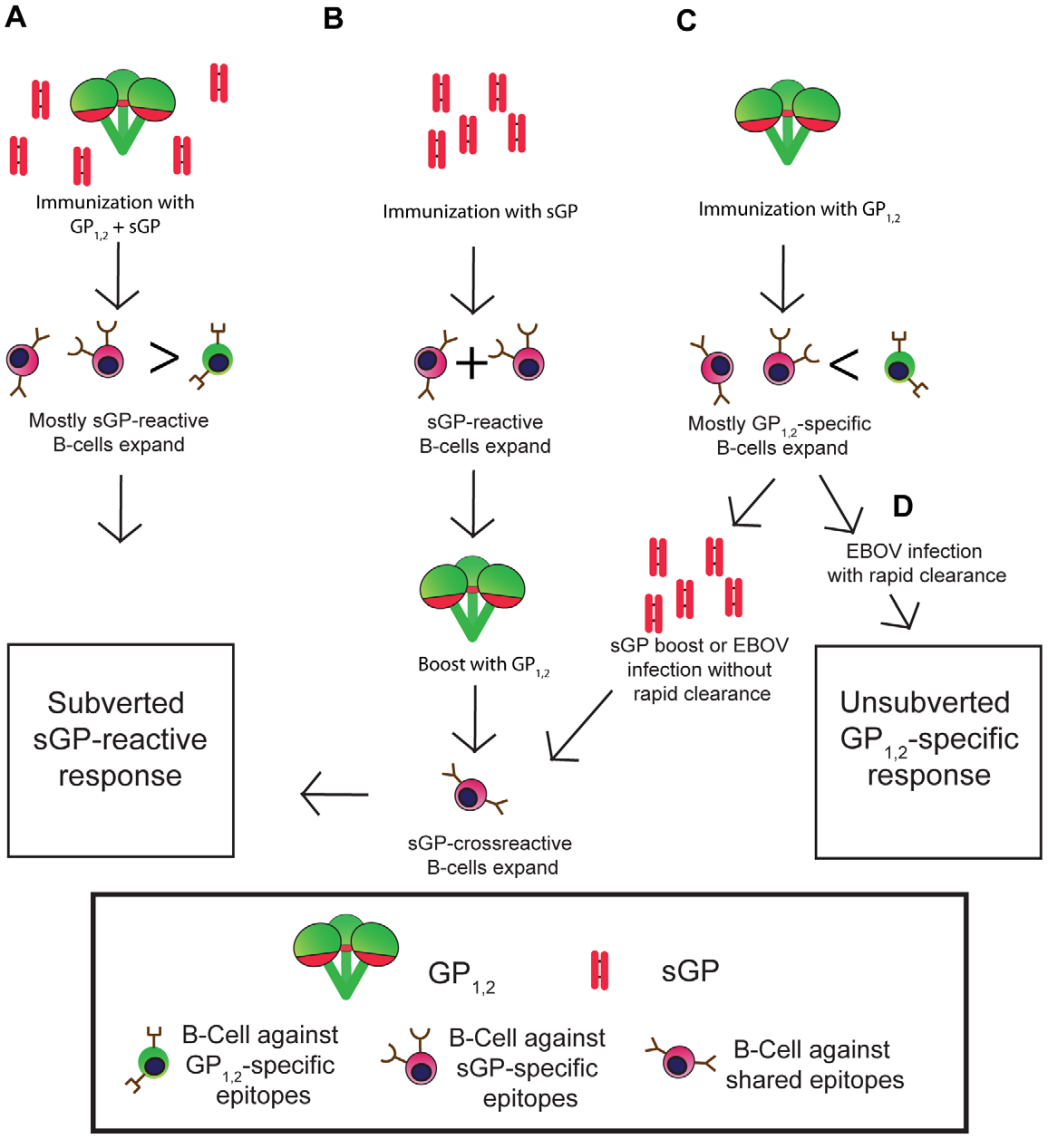
Proposed mechanism for antigenic subversion. Regions of GP_1,2_ that are shared with sGP are in red, while unshared epitopes are in green. B-cells are colored according to the regions of GP_1,2_ and sGP against which they react. (A) A naïve animal begins with B-cells that can potentially recognize epitopes distributed throughout GP_1,2_ and sGP. When sGP is expressed at much higher levels than GP_1,2_, as occurs during infection, those B-cells that recognize sGP epitopes, many of which are shared with GP_1,2_ (red regions of sGP and GP_1,2_) are preferentially activated and expanded compared to B-cells that recognize unshared epitopes of GP_1,2_ (green regions of GP_1,2_). Thus, sGP-reactive antibodies dominate the immune response. (B) Prior immunization by sGP. Because sGP shares over 90% of its linear sequence with GP_1,2_, animals primed with sGP generate anti-sGP antibodies, many of which are directed against epitopes shared with GP_1,2_. When these animals (or individuals who have previously been infected and recovered from EBOV infection) are boosted with GP_1,2_, sGP cross-reactive memory cells outnumber and express higher affinity receptors than naïve GP_1,2_ specific B-cells, resulting in preferential expansion of these sGP-cross-reactive B-cells and a predominantly sGP-reactive immune response. (C) Prior immunization by GP_1,2_. Priming naïve animals with GP_1,2_ results in antibodies largely against GP_1,2_ epitopes not shared with sGP, presumably due to the immunodominance and high accessibility of the GP_1,2_ mucin domain and shielding of shared epitopes. When these animals are boosted with sGP, or if they are infected with EBOV and do not have sufficiently high titers of anti-GP_1,2_ antibodies to clear the infection rapidly, memory B-cells that recognize shared epitopes encounter their cognate antigen and expand, while non-cross-reactive GP_1,2_-specific B-cells are not boosted, resulting in subversion of the host immune response towards sGP cross-reactivity. (D) Successful clearance of EBOV infection. In order to avoid sGP-mediated antigenic subversion, high enough titers of non-crossreactive anti-GP_1,2_ antibodies must be maintained to rapidly clear EBOV infection before subversion can occur.

The model we propose for the mechanism of antigenic subversion by sGP assumes that before immunization, the host begins with a repertoire of naïve B-cells that recognize epitopes distributed throughout GP_1,2_ and sGP (Fig. 8A). However, because sGP is generated in much higher quantities than GP_1,2_, B-cells that recognize sGP epitopes and epitopes shared between sGP and GP_1,2_ are more likely to encounter their cognate antigens as compared with B-cells that recognize GP_1,2_-specific epitopes. Furthermore, as the sGP-reactive B-cell population expands, it will outcompete other B-cells for antigen and survival signals. Thus, the humoral response is skewed towards sGP, and epitopes of GP_1,2_ that are shared with sGP. Antigenic subversion represents a novel viral escape strategy that has some similarities to original antigenic sin (OAS). In classical OAS, initial exposure to a pathogen results in a population of memory B-cells that recognize antigens specific to that pathogen strain. Upon subsequent exposure to a different strain of the same pathogen, cross-reactive memory B-cells will respond preferentially, producing antibodies with high affinity to the initial pathogen which may not bind to the new strain as effectively [37], [38]. Furthermore, these memory B-cells can compete for antigen and survival signals with naïve B-cells that might otherwise produce higher affinity or more protective antibodies to the new strain. Similarly, overexpression by Ebola virus of sGP ensures that sGP-reactive B-cells preferentially expand and outcompete GP_1,2_-specific B-cells for antigen and survival signals, resulting in a suboptimal host response that is directed away from membrane-bound GP_1,2_ on the virion surface. However, unlike classical OAS, this process does not require temporal separation of antigen encounters, but can also occur during simultaneous exposure to two partly identical antigens.

Our model for antigenic subversion can also explain how anti-GP_1,2_ antibodies from animals primed against sGP and then boosted with GP_1,2_ maintain cross-reactivity with sGP. In these animals, priming with sGP elicits antibodies against sGP epitopes, some of which are shared with GP_1,2_ (Fig. 8B). When these animals are boosted with GP_1,2_, memory B-cells that recognize shared epitopes vastly outnumber (and express higher affinity receptors than) the naïve B-cells that recognize unshared epitopes. Thus, the anti-sGP memory B-cells will be preferentially activated and expanded, boosting the anti-sGP response. This situation is analogous to one in which previously-infected individuals are vaccinated against GP_1,2_, and raises the possibility that immunizing such individuals may simply boost an already unprotective antibody response. While filovirus infection is rare, our findings suggest that it may be necessary to devise alternate strategies for immunizing previously-infected individuals in a way that specifically boosts the anti-GP_1,2_ response and avoids subversion.

Perhaps the most striking finding in this study is that boosting GP_1,2_-immunized mice with sGP could effectively subvert the anti-GP_1,2_ response and render it susceptible to competition by sGP. We hypothesize that while the majority of B-cells activated in mice immunized against GP_1,2_ are directed against epitopes not shared with sGP (Fig. 8C), there is a small population of activated B-cells that react with sGP. This is supported by our observation that even though sGP cannot measurably compete in ELISA and immunoprecipitation for anti-GP_1,2_ antibodies from GP_1,2_-immunized mice, these mice still develop low titers of sGP-binding antibodies. When GP_1,2_-immunized mice are boosted with sGP, these sGP-reactive B-cells expand while the remaining GP_1,2_-specific B-cells that recognize unshared epitopes do not, shifting the anti-GP_1,2_ antibody response from mostly GP_1,2_-specific to mostly sGP-cross reactive. Furthermore, it is notable that neutralizing activity actually decreased after boosting with sGP, despite an increase in overall anti-GP_1,2_ antibodies. Thus, boosting with sGP only augmented non-neutalizing anti-GP_1,2_ antibodies that are highly susceptible to sGP competition, while the existing neutralizing antibodies previously induced by GP_1,2_ in these mice maintained resistence to sGP interference. This situation is analogous to one in which an individual is immunized against GP_1,2_ is subsequently infected with EBOV. If the individual is unable to rapidly clear the virus, the virus may replicate sufficiently to subvert the host immune response. Thus, it will be critical for vaccines to induce high enough titers of anti-GP_1,2_ antibodies to ensure that the virus is cleared before it is able to effect subversion (Fig. 8D).

The inability of sGP to compete for anti-GP_1,2_ antibodies from GP_1,2_-immunized mice is consistent with a growing body of evidence pointing to the immunodominance of the GP_1,2_ mucin domain, a highly glycosylated region of GP_1_ not shared with sGP [24], [25]. This domain is thought to form a sterically bulky “cloak” that shields the putative receptor binding domain from host antibodies, as suggested for the HIV Env “glycan shield” [39]. The role that the mucin domain plays in host-pathogen interaction is complex and previous studies indicate that this region contains both neutralizing and infection-enhancing epitopes, and can mask epitopes on GP_1,2_ itself by steric occlusion [40], [41]. Furthermore, the mucin domain is the most divergent region of GP_1,2_ among EBOV strains, and is dispensible for GP_1,2_ mediated virus attachment and membrane fusion [42]–[44], strongly suggesting a role in protecting more functionally conserved regions of GP_1,2_ from immune attack. Because the linear sequence of sGP corresponds to the putative mucin-shielded receptor binding domain (RBD) of GP_1_, it is possible that sGP works together with the mucin domain so that host antibodies are directed either to shared epitopes that are sterically shielded in the GP_1,2_ trimer, or to the mucin domain itself, which is cleaved off in the host cell acidified endosome along with any bound antibodies [45], [46]. The possibility that GP_1,2_ epitopes shared with sGP may be shielded in the GP_1,2_ trimer is supported by our observation that very few anti-sGP antibodies in sGP-immunized mice cross-react with GP_1,2_ despite the fact that sGP shares over 90% of its linear sequence with GP_1,2_. Furthermore, antigenic subversion allows sGP to efficiently absorb those antibodies that do recognize unshielded and shared epitopes in GP_1,2_.

The importance of sGP-mediated antigenic subversion to EHF pathogenesis remains to be elucidated. Passive immunization studies with polyclonal sera or monoclonal antibodies will reveal whether sGP-crossreactive antibodies are in fact less protective than GP_1,2_-specific antibodies. This is particularly important given that passive transfer of anti-EBOV monoclonal antibodies has gained traction recently as a post-exposure therapeutic. If sGP cross-reactivity turns out to be correlated with impaired virus clearance, it would underscore the need to elicit and produce GP_1,2_-specific antisera or monoclonal antibodies for achieving more effective treatment of EBOV infection. Moreover, our findings also suggest that EBOV vaccines should be tailored to target regions not shared between sGP and GP_1,2_. This is particularly relevant to recent efforts to develop a broadly-protective vaccine, since these studies have centered around focusing vaccines on conserved epitopes by deleting highly variable regions of GP_1,2_ such as the mucin domain [24], [43], [47]. Because sGP actually corresponds to the most highly conserved region of GP_1_, antibodies elicited by these constructs may be cross-reactive with sGP and therefore susceptible to sGP-mediated subversion. Candidate pan-filovirus vaccines may need to be focused on regions of GP_1,2_ that are both highly conserved and unshared with sGP, such as the membrane-proximal GP_2_ subunit.

It will also be of great interest for EBOV vaccinology to determine whether antigenic subversion correlates with successes and failures of vaccines to protect animals against lethal challenge. It may be critical for an EBOV vaccine to elicit a long lasting immune response with high enough antibody titers so the host can clear the virus before it is able to replicate and effect antigenic subversion. This possibility is consistent with nonhuman primate lethal challenge experiments, in which survival was most closely correlated with maintenance of anti-GP_1,2_ antibody titers above a threshold level, while lower antibody titers only delayed the time to death [48]. Further, while much of EBOV vaccinology has focused on eliciting protective antibodies against the membrane-bound glycoprotein, a robust T-cell response may also improve vaccine efficacy. Immunization of nonhuman primates with a low dose of GP and nucleoprotein (NP)-expressing recombinant adenoviruses was demonstrated to elicit robust antibody and T-cell responses and confer protection against lethal challenge [49]. More importantly, EBOV-specific T-cells were shown to reduce the threshold of anti-GP_1,2_ antibodies needed for protection. Recombinant vectors expressing CTL epitopes have been demonstrated to confer protection to lethal EBOV challenge in mice, and GP-specific as well as nucleoprotein (NP)-specific CD8 T-cells can control infection even when adoptively transferred to otherwise naïve animals [50], [51]. These studies suggest that a robust T-cell response may reduce the threshold of antibodies needed for rapid virus clearance.

It is noteworthy that although the expression of sGP is conserved in Ebola viruses, sGP is not produced by Marburg virus (MARV), another member of the filoviridae. There are other instances where related viruses often diverge in the mechanisms they employ to survive in their respective hosts. For example, Sendai virus (SeV), a paramyxovirus that causes severe respiratory tract infections in rodents, expresses a V protein via RNA editing of the P gene. V is necessary for *in vivo* survival and pathogenesis of SeV, though V-deficient SeV show no defect in replication *in vitro* [52]. However, the closely related human parainfluenza virus type 1 (HPIV-1) does not express V, even though its P gene displays a high degree of homology to SeV P, and HPIV-1 causes similar disease in humans as SeV causes in rodents [53]. Similarly, while secretion of GP has not been observed in MARV, it has likely evolved alternative strategies to survive within its host.

While the precise relevance of antigenic subversion to Ebola vaccinology remains to be determined, antigenic subversion represents a novel and elegant solution to the challenge that viruses face of balancing the ability to infect host cells efficiently while evading host immune surveillance. The constraints of a very small genome neccessitate packing a great deal of functionality into a small space, and sGP-mediated subversion represents a mechanism which, along with glycan-dependent steric shielding, and immunodominance of the GP_1,2_ mucin domain, may help EBOV to survive in its host. Improving our understanding of how these mechanisms work together will eventually open the door to a more rationally designed vaccine. A vaccine directed against highly conserved regions of GP_1,2_, such as the GP_2_ subunit, could induce broadly reactive antibodies while also avoiding the potential for sGP-mediated immune subversion. Such a vaccine could protect against multiple strains of EBOV, including strains that have not yet been identified.

## Materials and Methods

### Ethics Statement

This study was carried out in strict accordance with the recommendations in the Guide for the Care and Use of Laboratory Animals of the National Institutes of Health. Animal ethics approval for the immunization studies in mice was obtained from the Institutional Animal Care and Use Committee (IACUC) at Emory University. All animal studies were performed under approval from the Institutional Animal Care and Use Committee (IACUC) at Emory University. Female BALB/c mice (8-week old) were purchased from the Jackson Laboratory and housed in the animal facility at the Emory University.

### Cell Lines and Plasmids

293T cells and HeLa cells were maintained in Dulbecco's Modified Eagle's Medium (DMEM, Mediatech) supplemented with 10% fetal bovine serum (Hyclone, ThermoFisher) and penicillin/streptomycin. All Ebola glycoprotein constructs were based on the Ebola Zaire strain (ZEBOV), Mayinga Subtype (GenBank accession# U23187.1). Editing site mutants were generated in pBlueScript II K/S+ vector through site-directed mutagenesis using the QuickChange XL kit (Stratagene). Constructs were then subcloned pCAGGS mammalian expression vector. Protein expression was carried out by transfecting 90% confluent cells in 6-well plates with 5 µg DNA+12 µL Fugene HD (Roche) per well, as per manufacturer instructions, and detected at 48 h post transfection. Surface expression was detected by surface biotinylation followed by immunoprecipitation with anti-EBOV GP mouse polyclonal antibody, SDS-PAGE, and Avidin-HRP blotting. Cell lysate was harvested in cell lysis buffer and cell culture supernatant was collected, spun down to remove cell debris, and concentrated 10× by a centrifugal concentrator. Cell lysate and concentrated cell culture supernatant were run on SDS-PAGE under denaturing conditions, followed by probing with anti-EBOV GP_1,2_/sGP rabbit polyclonal antibody.

### Vaccine Preparation and Immunization

Mutant ZEBOV GP plasmids for DNA immunization experiments were prepared using the EndoFree Plasmid Mega Kit (Qiagen) as per manufacturer instructions and redissolved in pure endotoxin-free water at a concentration of 4–6 µg/µL, and purity was verified by restriction analysis and spectrophotometry. For immunization, DNA was diluted in sterile PBS to 0.5 µg/µL and filter sterilized. Female BALB/C mice (Charles River Laboratory) at six mice per group received 50 µg of DNA intramuscularly (25 µg/leg) per immunization. Anesthetized mice were bled retro-orbitally two weeks after each immunization and serum samples were stored at −80°C until use.

### Recombinant Protein Production and ELISA

Production of purified histidine-tagged HA has been described previously [54]. Soluble histidine-tagged GP_1,2_ and sGP were generated by C-terminal addition of a single 6× histidine tag. Soluble GP_1,2_ was generated by truncation of the transmembrane domain and cytoplasmic tail. Recombinant vaccinia viruses (rVV) were generated as described elsewhere to synthesize soluble His-tagged GP_1,2_ (His- GP_1,2_) and sGP (His-sGP), as well as membrane-bound GP_1,2_ [55]. For production and purification of His-GP_1,2_ and His-sGP, rVV-infected cell supernatant was clarified and purified using a PrepEase His Purification Kit (Affymetrix) and purity of recombinant protein was verified by SDS-PAGE followed by Western blot or coomassie stain. Further, purified His-GP_1,2_ and His-sGP were tested for reactivity to pre-immune sera or sera from unvaccinated mice by ELISA and Western blot, and they were found to be unreactive. For ELISA, flat-bottom Immulon 4-HBX 96-well plates (Thermo) were coated overnight with 0.1 µg/well of His- GP_1,2_ or His-sGP. A standard curve was generated by coating control wells with known concentrations of mouse IgG. Plates were washed 5× in PBS+Tween (PBST), blocked in PBST+2%BSA, and then incubated in duplicate for two hours with antisera diluted in PBST+2%BSA. Plates were washed again, and incubated with 1∶1000 (pooled anti- IgG subtype) HRP-conjugated goat anti-mouse secondary antibody. After final wash, plates were developed with 3,3′,5,5′-Tetramethylbenzidine (TMB, Thermo) and stopped at 5 minutes with 0.2 M HCl. Plates were read and antibody concentration was calculated using the standard curve.

### Competition ELISA

Competition ELISA was performed by modifying the above protocol. Plates were coated with His- GP_1,2_. Pooled antisera were diluted in PBST+2%BSA to a concentration corresponding to an OD of 1.0 by anti- GP_1,2_ ELISA. Diluted antisera were then mixed with decreasing concentrations of purified His-sGP or His- GP_1,2_ and immediately added to His- GP_1,2_-coated wells. The ELISA was then developed as described above and competition was calculated as percent of signal compared to no competing antigen.

### Competition Immunoprecipitation

Competition immunoprecipitation was performed by incubating pooled antisera (normalized for anti-GP_1,2_ titer as determined by ELISA) with 200 ng of purified His- GP_1,2_ and increasing amounts purified His-sGP at molar ratios of 0.25∶1, 1∶1, 4∶1, and 8∶1 sGP∶GP_1,2_. Antisera incubated with His-sGP alone, His-GP_1,2_ alone, or with no GP were used as controls, as well as antisera incubated with GP_1,2_ in the presence of recombinant influenza HA. Samples were incubated on ice for 20 minutes, followed by addition of protein-G coupled agarose beads (Thermo Scientific) to further incubate at 4°C for an additional two hr with agitation. Samples were then centrifuged and washed three times with with lysis buffer, and then mixed with 6× Laemmli SDS sample buffer with 12% β-mercaptoethanol. The samples were heated at 95°C for 5 minutes and then used for SDS-PAGE followed by Western blot analysis using antibodies gainst both sGP and GP_1,2_.

### Affinity of Polyclonal Antisera

Apparent affinity of polyclonal antisera was determined by quantitative ELISA using purified IgG from immunized animals. IgG was purified using Melon Gel (Thermo) as per manufacturer instructions and purity of IgG was verified by ELISA and coomassie gel staining. Since quantitative affinity ELISA requires that coating antigen be incubated with increasing dilutions of antibodies until coating antigen becomes saturated, we found that high antibody concentrations can result in signals that exceed the plate reader's range of detection. Thus, we titrated the amount of coating antigen down to 0.05 µg/well to avoid signal saturation. Wells were coated overnight with 0.05 µg of purified His-GP_1,2_ or His-sGP and after washing and blocking were incubated with purified IgG diluted in PBST+2%BSA, at dilutions ranging from 1∶10 to 1∶1280 (based on original serum volume). ELISAs were developed as described above and the signal converted to nM concentration of IgG by comparison to a standard curve. Apparent K_d_'s of polyclonal sera were calculated by nonlinear regression analysis using GraphPad Prism. These results were verified manually by analysis of linearized binding curves as detailed elsewhere [33].

### Pseudovirus Generation and Neutralizing Assay

EBOV-GP pseudotyped HIV was generated as described elsewhere [56]. Briefly, 293T-cells were cotransfected with Env-defective HIV backbone and ZEBOV GP in pCAGGS vector using Fugene HD (Roche). Supernatants were harvested 48 h post-transfection, clarified, and filtered using a 0.45 micron filter. Pseudoviruses were titered by infecting JC53 cells [57], which express β-galactosidase and luciferase under a *tat-*activated promoter, causing infected cells turning blue with X-Gal staining. Neutralization assays were performed as described elsewhere [56] with minor modifications. Briefly, pseudoviruses were pre-incubated with dilutions of heat-inactivated antisera, and supplemented with heat-inactivated naïve mouse sera (Innovative Research) so that 5% of the total volume was mouse serum. Pseudovirus-antiserum mixtures were then added to 30% confluent JC53 cells and incubated for 48 h. Virus infection and neutralization was measured by luciferase reporter assay, and neutralization was measured by decrease in luciferase expression compared to virus-only controls [57].

We performed a competition neutralization assay by selecting a fixed antisera concentration corresponding to either 50% or 80% neutralizing activity. Diluted antisera were incubated with dilutions of purified His-sGP or with soluble influenza PR8 hemagluttinin (HA) as a control (GenBank Accession# JF690260). Antisera mixtures were then mixed with pseudovirus and the neutralization assay was developed as described above. Interference with neutralization was determined by the percent rescue of infectivity compared to wells with pseudovirus+antisera without competing sGP, as calculated by the formula [(virus+antibody+sGP)−(virus+antibody)]/[(virus alone)−(virus+antibody)]×100.

## Supporting Information

Figure S1 **Competition cell surface ELISA.** HeLa cells were seeded in a 96-well plate and allowed to grow overnight to 100% confluency. Cells were then infected at an MOI of 5 with a recombinant vaccinia virus that directs infected cells to express membrane-bound EBOV GP_1,2_. At 24 h post-infection, cells were fixed in 2% paraformaldehyde and washed in PBS. Pooled antisera from mice immunized with sGPEdit (light red), GP-7A (dark red), GP-8A (light blue), or GP_1,2_Edit (dark blue) were diluted to give roughly equivalent anti-GP_1,2_ signal. Diluted antiserum was mixed with increasing quantities of purified his-sGP and incubated with fixed GP_1,2_ expressing cells for two hours to allow sGP to compete with GP_1,2_ for antibodies. ELISAs were developed as previously described with the exception that detergent-free PBS was used in washing steps.
(TIF)

Figure S2 **Interference with antibody-mediated neutralization by sGP at 50% neutralizing activity.** The ability of sGP to interfere with antibody-dependent neutralization was determined identically to Figure 4B, except that the concentration of antisera was fixed to correspond to 50% neutralization. Pooled GP_1,2_-immunized (blue) and sGP-immunized (red) antisera were co-incubated with increasing dilutions of his-sGP (solid markers) or his-influenza PR8 HA (open markers), and rescue of infectivity was measured as described in methods.
(TIF)

Figure S3 **Expression of GP_1,2_ and sGP together.** Because antigen expression from DNA vaccines is too low to detect *in vivo*, we measured expression in cell culture as a proxy for *in vivo* expression. HeLa cells in 6-well plates were transfected with GP_1,2_Edit, sGPEdit, and empty pCAGGS vector at the same ratio as used to immunize animals and 5 µg total DNA per well. Expression of sGP and GP_1,2_ was determined 36 h post-transfection in both cell lysate and culture supernatant by Western blot using a polyclonal rabbit antibody that reacts with both GP isoforms. The volume of cell lysate and supernatant analyzed for each sample was proportional to the total amount of lysate and supernatant collected so that the Western blots reflect the relative amounts of total sGP and GP_1,2_ produced.
(TIF)

Figure S4 **Immunization with lower ratios of sGP∶GP_1,2_.** Female BALB/C mice were immunized IM with 50 µg of total DNA per immunization as in previous immunization experiments and boosted at week 4. The amount of GP_1,2_Edit was fixed at 12.5 µg, and groups were immunized with 1∶1, 1∶3, and 1∶9 ratios of sGP Edit∶GP_1,2_ Edit, as well as GP_1,2_Edit without sGPEdit. Total immunizing DNA was normalized to 50 µg with empty pCAGGS vector. (Top Panel) sGP competition ELISA. Pooled antisera were analyzed from immunized mice at week 6 and the ability of sGP to compete for anti-GP_1,2_ antibodies was determined by competition ELISA as described in Figure 3B. (Bottom Panel) *In Vitro* antigen expression. HeLa cells were transfected with GP_1,2_Edit, sGPEdit, and empty pCAGGS vector at the same ratio as used to immunize animals and 5 µg total DNA per well. Expression of sGP and GP_1,2_ was determined 36 h post-transfection as describe in Figure S3. Both cell lysate and culture supernatant were analyzed by Western blot using a polyclonal rabbit antibody that reacts with both GP isoforms.
(TIF)

Figure S5 **Interference with antibody-mediated neutralization by sGP at 50% neutralizing activity from GP_1,2_+sGP antisera.** The ability of sGP to interfere with antibody-dependent neutralization was determined identically to Figure 6F, except that the antiserum concentration was fixed to correspond to 50% neutralization. Pooled GP_1,2_+sGP-immunized antisera were co-incubated with increasing dilutions of sGP (red) or influenza PR8 HA (blue), and rescue of infectivity was measured as described in methods.
(TIF)
