# Supplementary figures and images for: Antigenic Subversion: A Novel Mechanism of Host Immune Evasion by Ebola Virus
Source: PLoS Pathog. 2012 Dec 13;8(12):e1003065. doi: 10.1371/journal.ppat.1003065 (PMC3521666; doi:10.1371/journal.ppat.1003065)

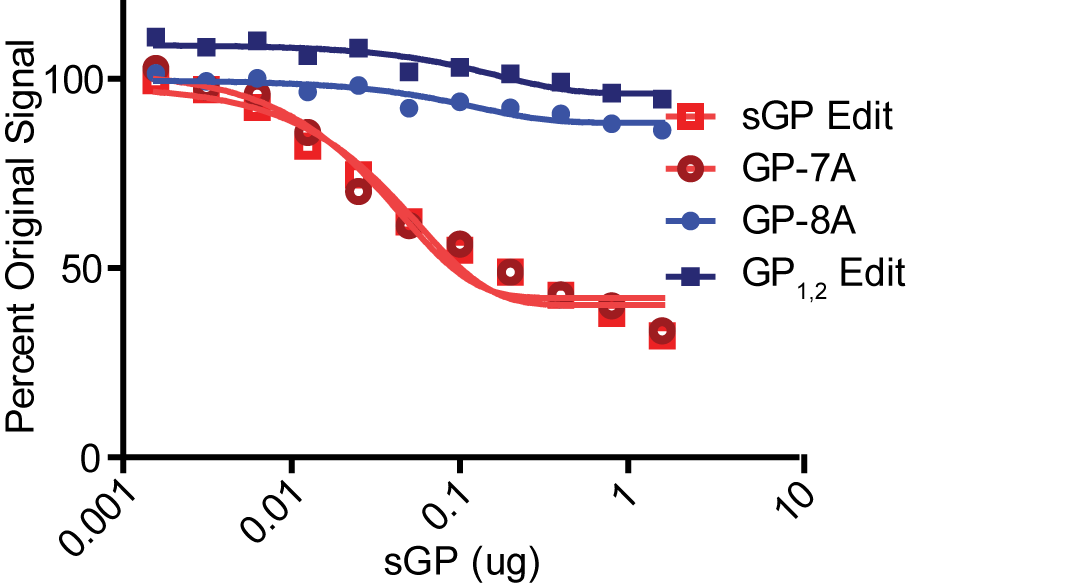

Supplement: Figure S1 — Competition cell surface ELISA. HeLa cells were seeded in a 96-well plate and allowed to grow overnight to 100% confluency. Cells were then infected at an MOI of 5 with a recombinant vaccinia virus that directs infected cells to express membrane-bound EBOV GP1,2. At 24 h post-infection, cells were fixed in 2% paraformaldehyde and washed in PBS. Pooled antisera from mice immunized with sGPEdit (light red), GP-7A (dark red), GP-8A (light blue), or GP1,2Edit (dark blue) were diluted to give roughly equivalent anti-GP1,2 signal. Diluted antiserum was mixed with increasing quantities of purified his-sGP and incubated with fixed GP1,2 expressing cells for two hours to allow sGP to compete with GP1,2 for antibodies. ELISAs were developed as previously described with the exception that detergent-free PBS was used in washing steps. (TIF) [file ppat.1003065.s001.tif]

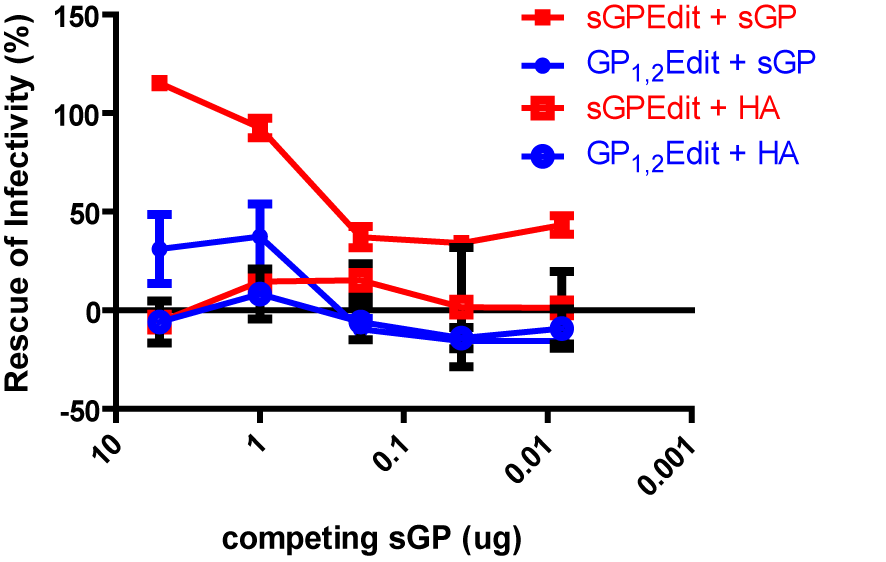

Supplement: Figure S2 — Interference with antibody-mediated neutralization by sGP at 50% neutralizing activity. The ability of sGP to interfere with antibody-dependent neutralization was determined identically to Figure 4B, except that the concentration of antisera was fixed to correspond to 50% neutralization. Pooled GP1,2-immunized (blue) and sGP-immunized (red) antisera were co-incubated with increasing dilutions of his-sGP (solid markers) or his-influenza PR8 HA (open markers), and rescue of infectivity was measured as described in methods. (TIF) [file ppat.1003065.s002.tif]

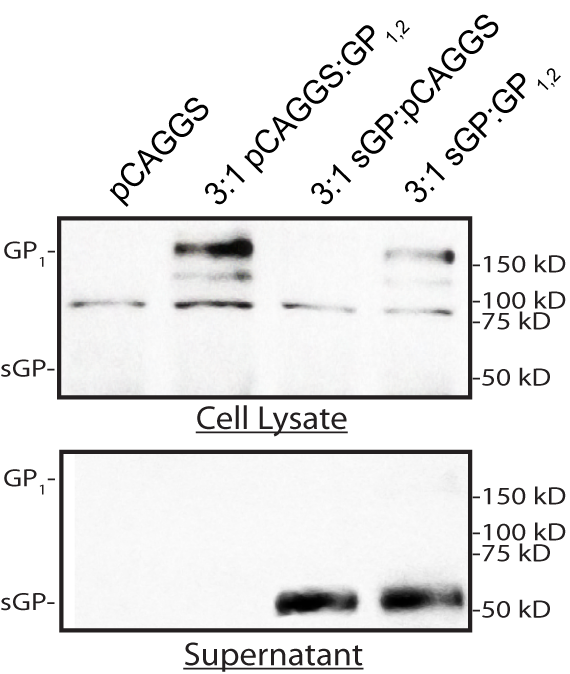

Supplement: Figure S3 — Expression of GP1,2 and sGP together. Because antigen expression from DNA vaccines is too low to detect in vivo, we measured expression in cell culture as a proxy for in vivo expression. HeLa cells in 6-well plates were transfected with GP1,2Edit, sGPEdit, and empty pCAGGS vector at the same ratio as used to immunize animals and 5 µg total DNA per well. Expression of sGP and GP1,2 was determined 36 h post-transfection in both cell lysate and culture supernatant by Western blot using a polyclonal rabbit antibody that reacts with both GP isoforms. The volume of cell lysate and supernatant analyzed for each sample was proportional to the total amount of lysate and supernatant collected so that the Western blots reflect the relative amounts of total sGP and GP1,2 produced. (TIF) [file ppat.1003065.s003.tif]

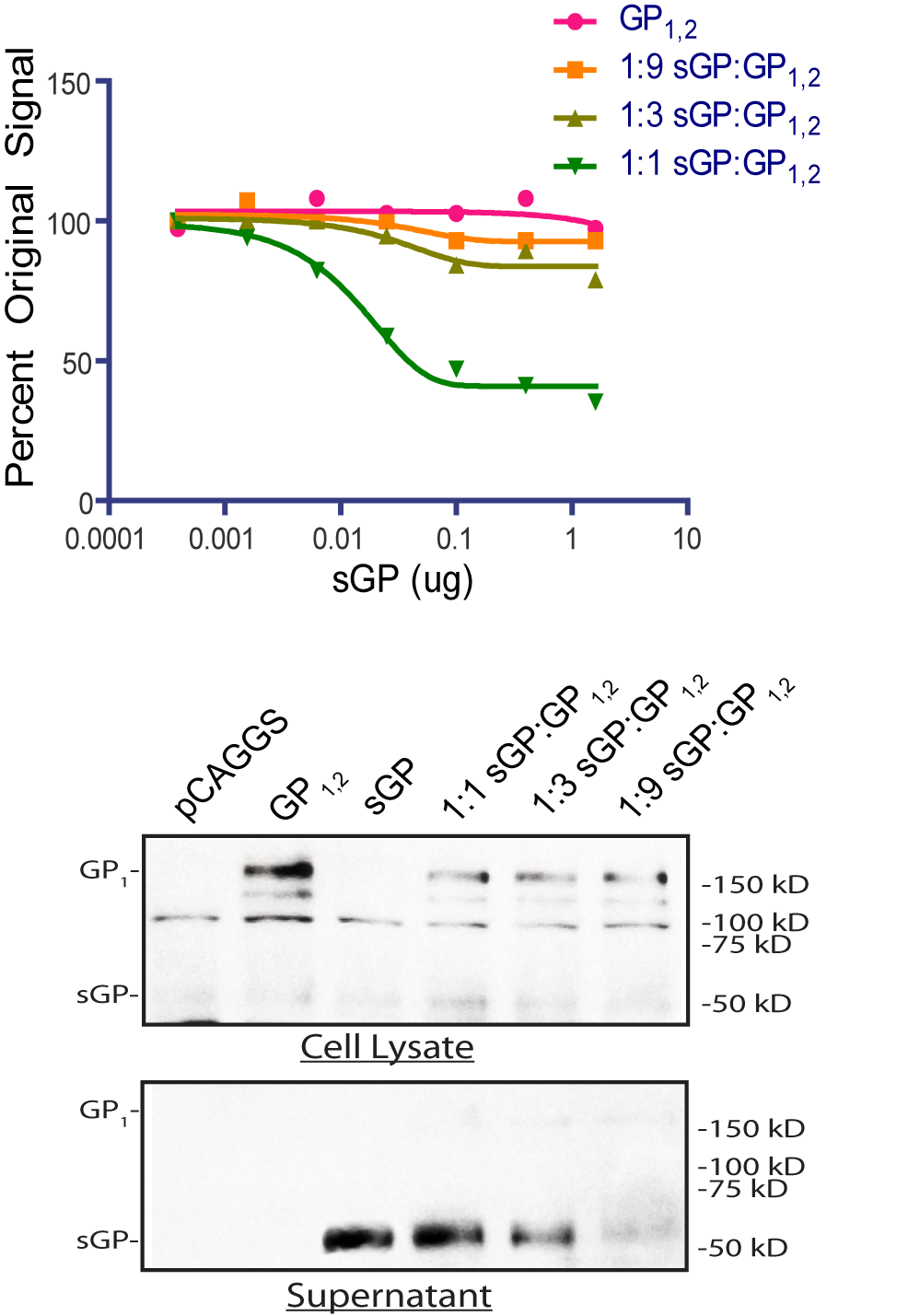

Supplement: Figure S4 — Immunization with lower ratios of sGP∶GP1,2. Female BALB/C mice were immunized IM with 50 µg of total DNA per immunization as in previous immunization experiments and boosted at week 4. The amount of GP1,2Edit was fixed at 12.5 µg, and groups were immunized with 1∶1, 1∶3, and 1∶9 ratios of sGP Edit∶GP1,2 Edit, as well as GP1,2Edit without sGPEdit. Total immunizing DNA was normalized to 50 µg with empty pCAGGS vector. (Top Panel) sGP competition ELISA. Pooled antisera were analyzed from immunized mice at week 6 and the ability of sGP to compete for anti-GP1,2 antibodies was determined by competition ELISA as described in Figure 3B. (Bottom Panel) In Vitro antigen expression. HeLa cells were transfected with GP1,2Edit, sGPEdit, and empty pCAGGS vector at the same ratio as used to immunize animals and 5 µg total DNA per well. Expression of sGP and GP1,2 was determined 36 h post-transfection as describe in Figure S3. Both cell lysate and culture supernatant were analyzed by Western blot using a polyclonal rabbit antibody that reacts with both GP isoforms. (TIF) [file ppat.1003065.s004.tif]

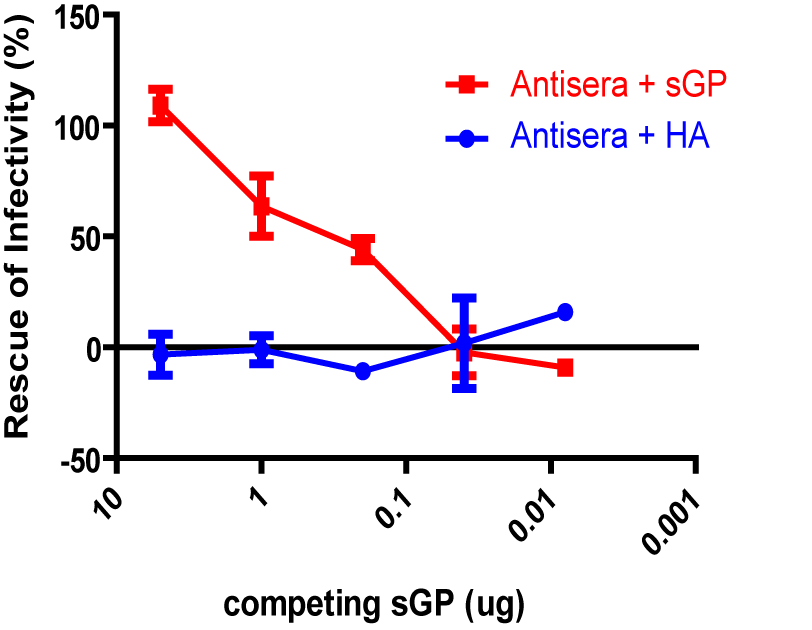

Supplement: Figure S5 — Interference with antibody-mediated neutralization by sGP at 50% neutralizing activity from GP1,2+sGP antisera. The ability of sGP to interfere with antibody-dependent neutralization was determined identically to Figure 6F, except that the antiserum concentration was fixed to correspond to 50% neutralization. Pooled GP1,2+sGP-immunized antisera were co-incubated with increasing dilutions of sGP (red) or influenza PR8 HA (blue), and rescue of infectivity was measured as described in methods. (TIF) [file ppat.1003065.s005.tif]
